# Arthroscopic Tibial Tubercle Osteophyte Debridement and Gout Crystal Clearance for the Treatment of Osgood-Schlatter Disease Complicated With Gout in Patients With Anterior Knee Pain

**DOI:** 10.1016/j.eats.2024.103369

**Published:** 2025-01-03

**Authors:** Zhen-long Liu, Ling-jie Chen, Yu-chen Qiu, Yue-yang Hou, Zhi-hui He, Jian-li Gao, Hong-shi Huang, Yu-ping Yang

**Affiliations:** a Peking University Health Science Center, Beijing, P. R. China; b Department of Sports Medicine, Peking University Third Hospital, Beijing, P. R. China; c Key Laboratory of Sports Injuries, Beijing, P. R. China

## Abstract

We describe the arthroscopic tibial tubercle osteophyte debridement and gout crystal clearance for treating Osgood-Schlatter disease combined with gout in patients experiencing anterior knee pain. Preoperative assessment includes medical history review, physical examination, and imaging studies. The surgical procedure involves arthroscopic entry, osteophyte and calcification clearance, and crystal removal. Postoperative rehabilitation allows for full weightbearing, with moderate knee flexion exercises. This approach offers a promising treatment option for patients with Osgood-Schlatter disease and gout, providing new insights into surgical management strategies.

Osgood-Schlatter disease (OSD) is a common condition causing anterior knee pain in adolescents and adults. This disease typically occurs during the early adolescent growth spurt between the ages of 10 and 15.^1^^,^^2^ Symptoms often persist until the end of the growth spurt, but studies have shown that OSD symptoms may persist into adulthood and affect motor function and quality of life. For severe or refractory cases, surgical intervention may be considered.^3^

Gout is a crystal-related joint disease caused by monosodium urate deposition in the joints, often accompanied by hyperuricemia. Studies have indicated that tendons are the most common sites for crystal deposition, with the patellar tendon being the most affected site.^4^^,^^5^ Treatment for gout includes controlling uric acid levels, pain management, and other conservative measures. Surgical intervention may be considered for patients with large gout stones, chronic unresolved pain, or severe skin breakdown.^6^

We describe the use of arthroscopic tibial tubercle osteophyte debridement combined with gout crystal clearance for the treatment of OSD complicated with gout in patients with anterior knee pain.

## Surgical Technique

### Preoperative Evaluation

**Fig 1:**
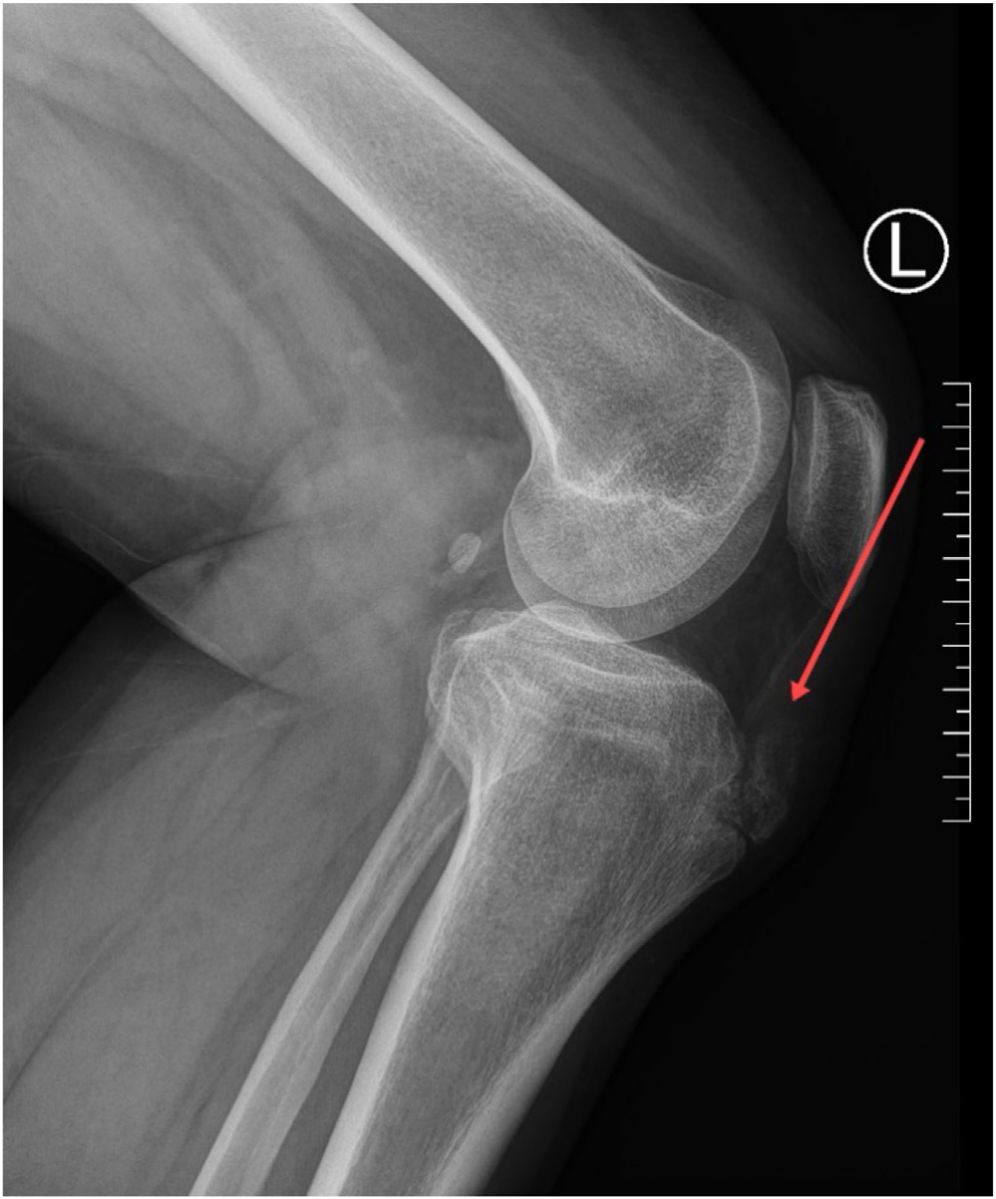
The sagittal view of the x-ray of the patient’s left knee. The red arrow points to the tibial tubercle osteophyte, consistent with the characteristics of Osgood-Schlatter disease.

**Fig 2:**
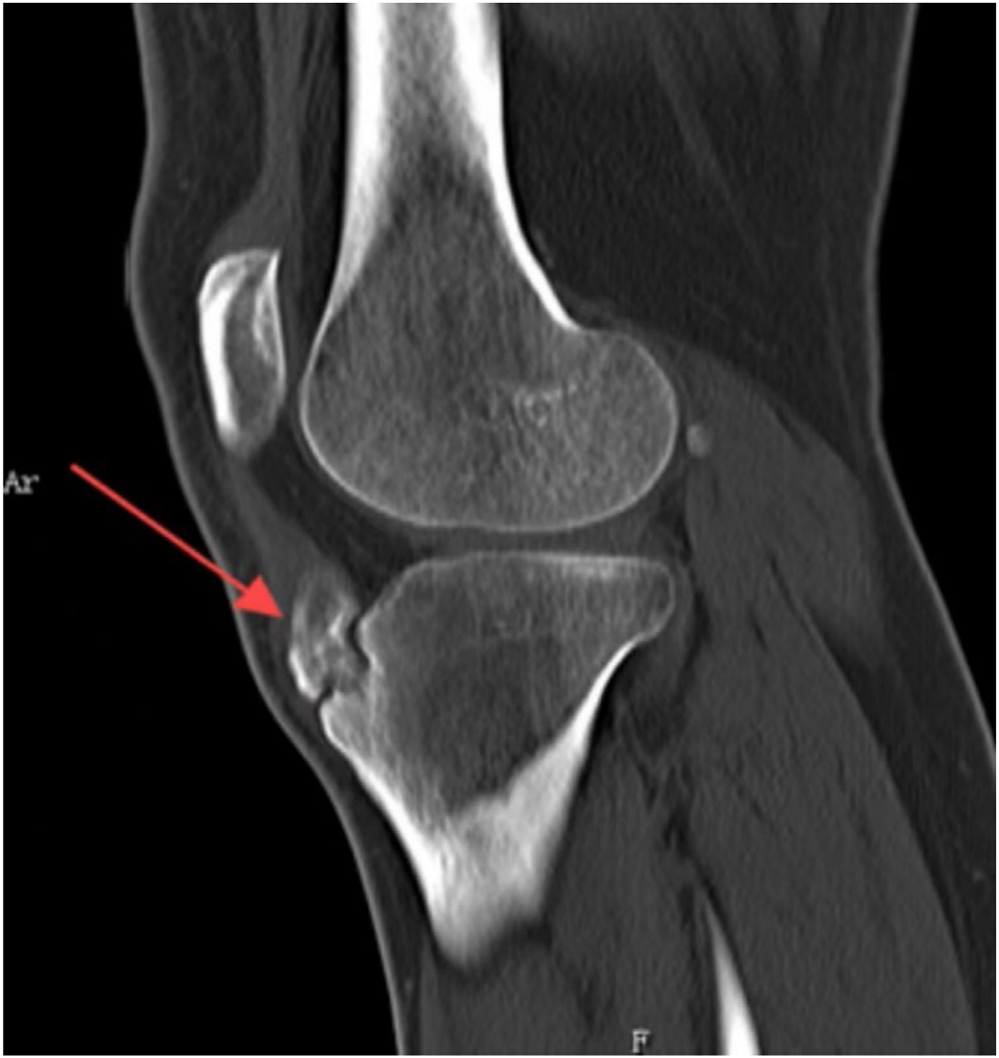
The sagittal view of the computed tomography scan of the patient’s left knee. The red arrow points to the tibial tubercle osteophyte, consistent with the characteristics of Osgood-Schlatter disease.

**Fig 3:**
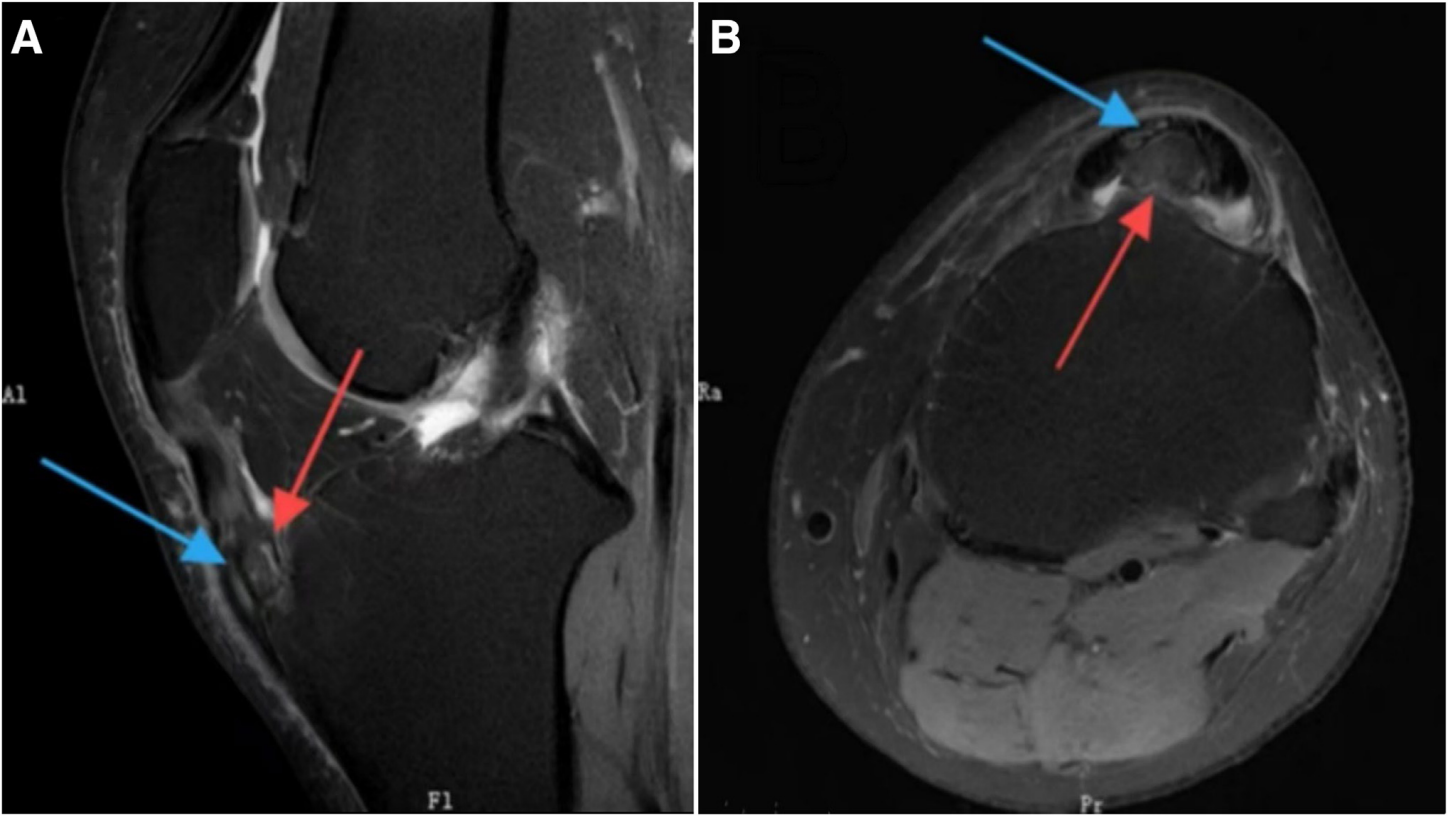
The view of the magnetic resonance imaging of the patient’s left knee. The area pointed by the red arrow is the tibial tuberosity osteophyte, and the area pointed by the blue arrow is damage at the insertion of the patellar tendon. Image A is in the sagittal plane, and image B is in the axial plane.

Preoperative evaluation includes reviewing medical history, physical examination, specialized imaging studies, and routine preoperative assessments. Based on magnetic resonance imaging (MRI), x-ray, and computed tomography (CT) scans (Fig 1, Fig 2, Fig 3), the location and size of the tibial tubercle osteophyte need to be confirmed, as well as whether the lesion involves the patellar tendon and the presence of intra-articular lesions causing anterior knee pain. In patients with concomitant gout, because MRI, x-ray, and CT scans have low detection rates for urate crystal examination, arthroscopic exploration during surgery can be performed to clear gout crystals.

### Preoperative Preparation

After anesthesia, the patient lies supine on the operating table, with the surgical leg hanging by the side of the table, while the nonsurgical leg is placed flat on the table to avoid interfering with the surgical procedure. A proximal thigh tourniquet is applied and inflated to a pressure of 40 kPa during surgery.

### Surgical Procedure

**Fig 4:**
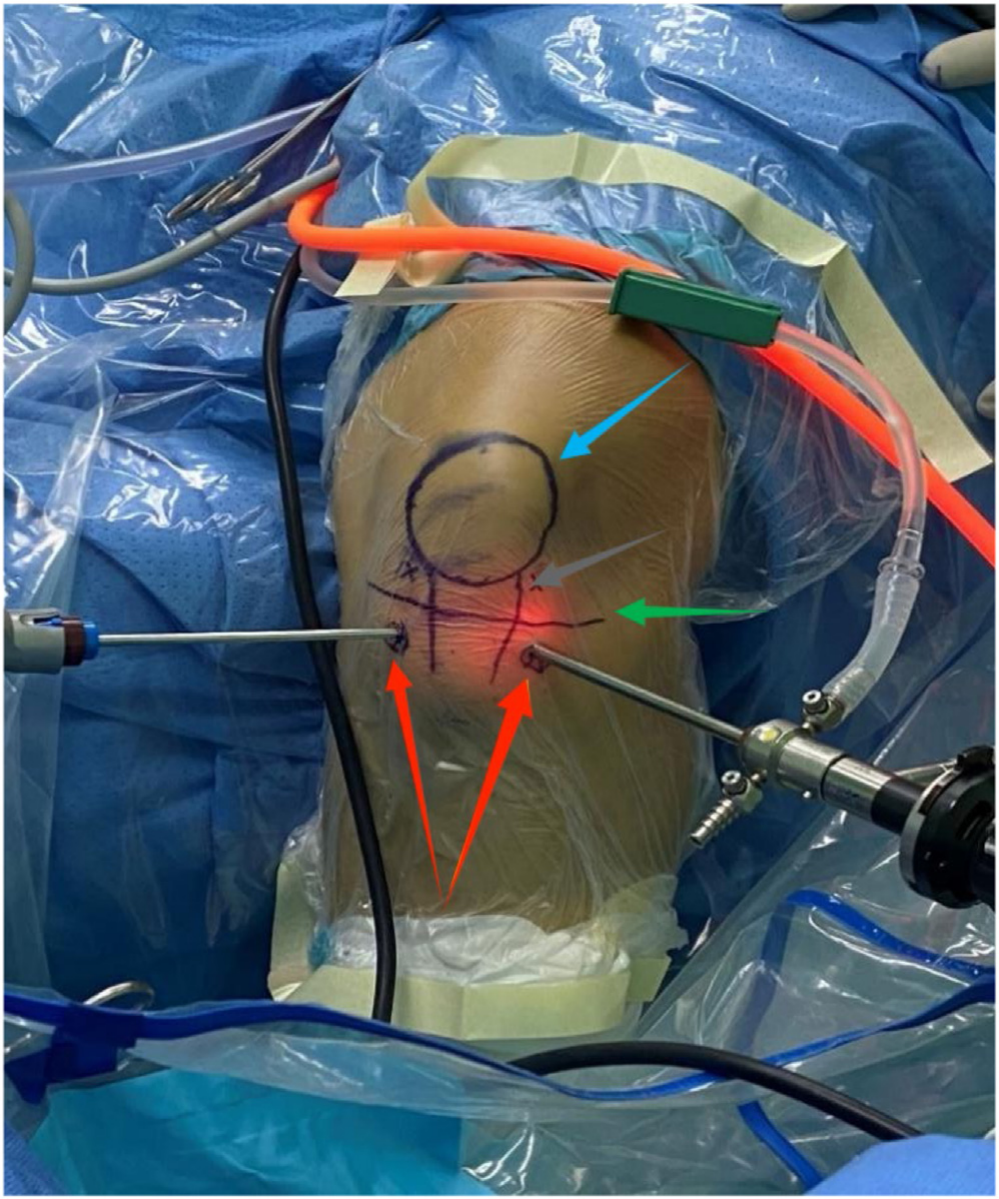
Surgical portal illustration. The surface positions of the lateral portal to the tibial tubercle and the medial portal to the tibial tubercle are about 2 cm beneath the joint space (bony landmark of the tibial tubercle) and approximately 1.5 cm from the edge of the patellar tendon. The blue row points to the patella location. The gray row points to the edge of the patellar tendon location. The green row points to the joint space location. The red row points to 2 portal locations.

The first step is to locate the portal position. The surface positions of the lateral portal to the tibial tubercle and the medial portal to the tibial tubercle are about 2 cm beneath the joint space (bony landmark of the tibial tubercle) and approximately 1.5 cm from the edge of the patellar tendon (Fig 4). The second step is to explore the surgical field.

**Fig 5:**
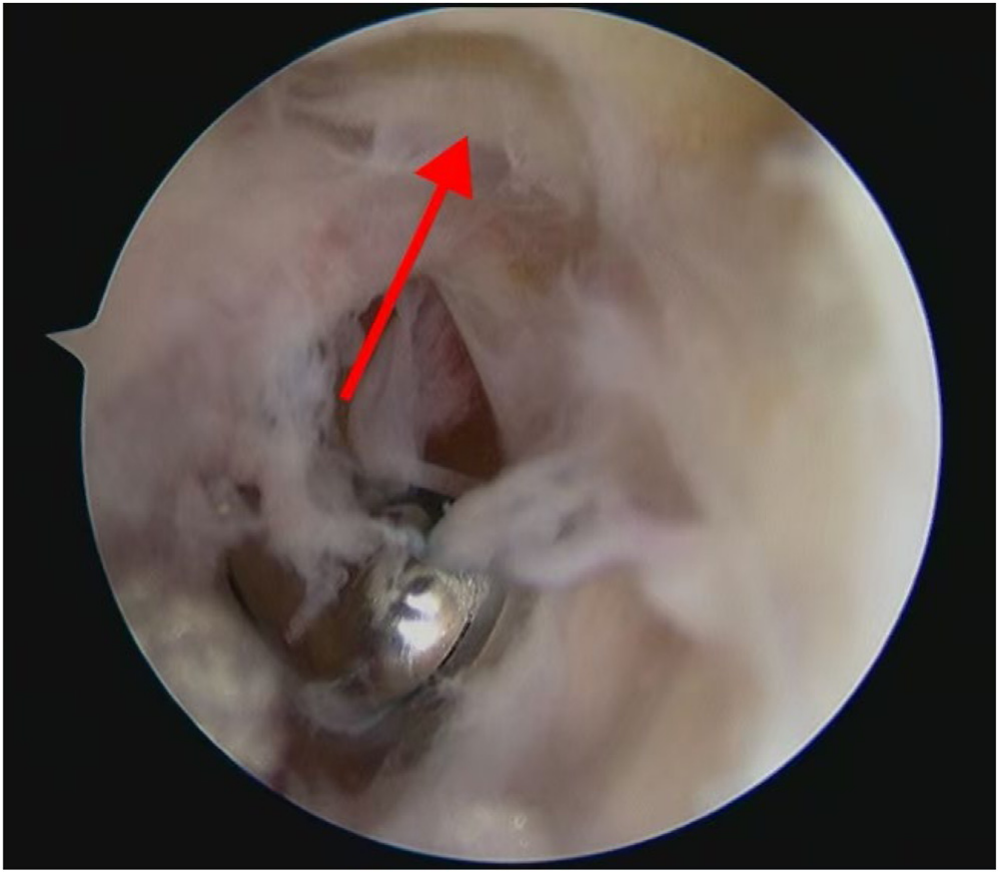
Arthroscopic view of the patient’s left knee through the lateral portal to the tibial tubercle. The area pointed to by the arrow is the infrapatellar fat pad.

**Fig 6:**
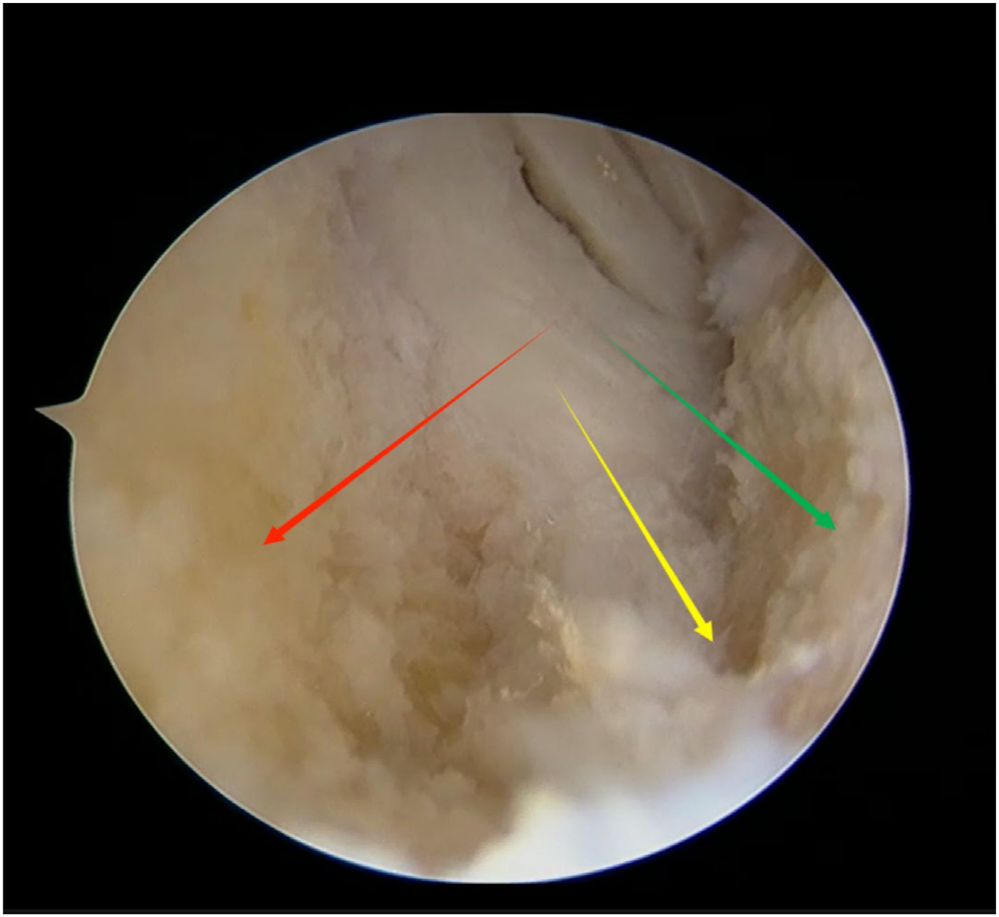
Arthroscopic view of the patient’s left knee through the lateral portal to the tibial tubercle. The red arrow points to the posterior side of the patellar tendon, the green arrow points to the tibial tubercle osteophyte, and the blue arrow points to the proximal patellar tendon insertion.

**Fig 7:**
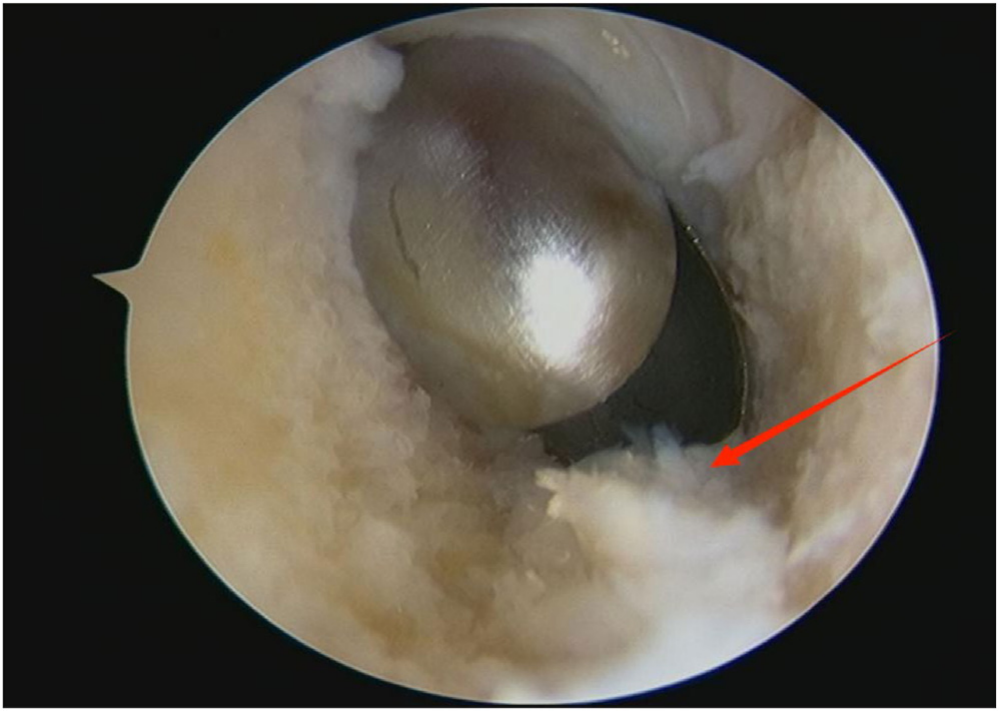
Arthroscopic view of sampling of a portion of the tibial tubercle osteophyte for pathological examination through the medial portal to the tibial tubercle. The red arrow points to the a portion of the tibial tubercle osteophyte.

**Fig 8:**
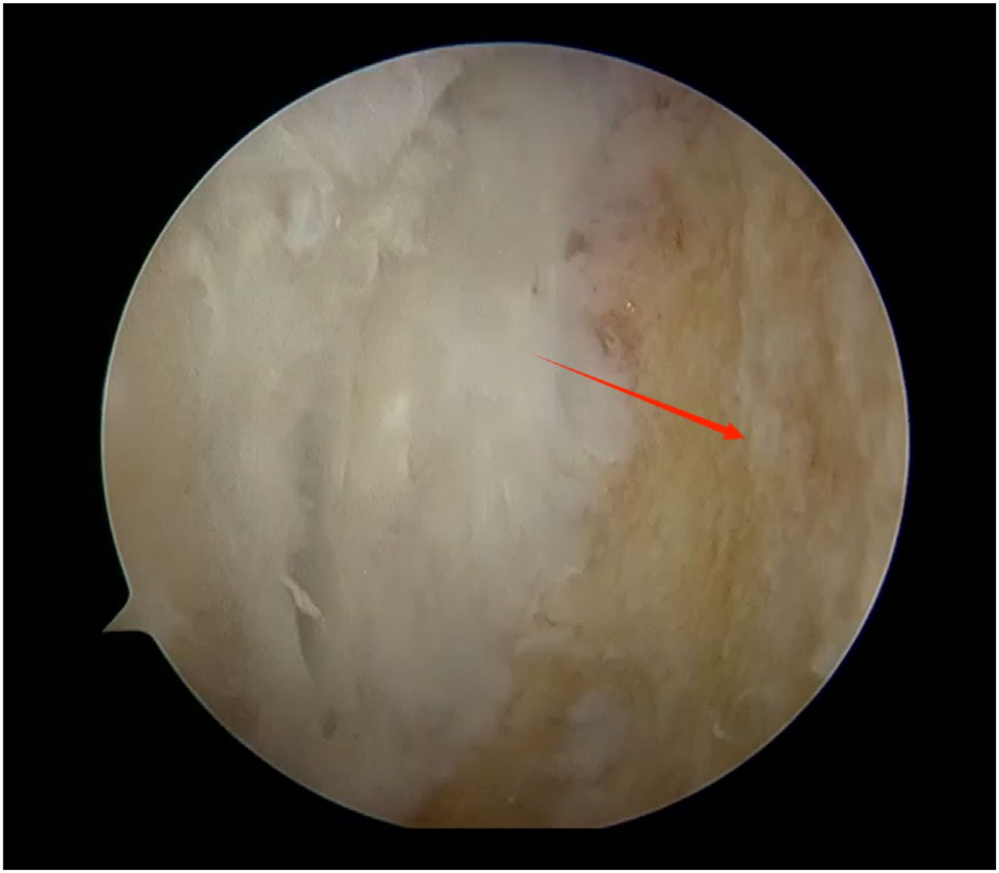
Arthroscopic view of the patient’s left knee through the lateral portal to the tibial tubercle. The red arrow indicates that the protruding part of the tibial tuberosity has disappeared, leaving a slight depression.

**Fig 9:**
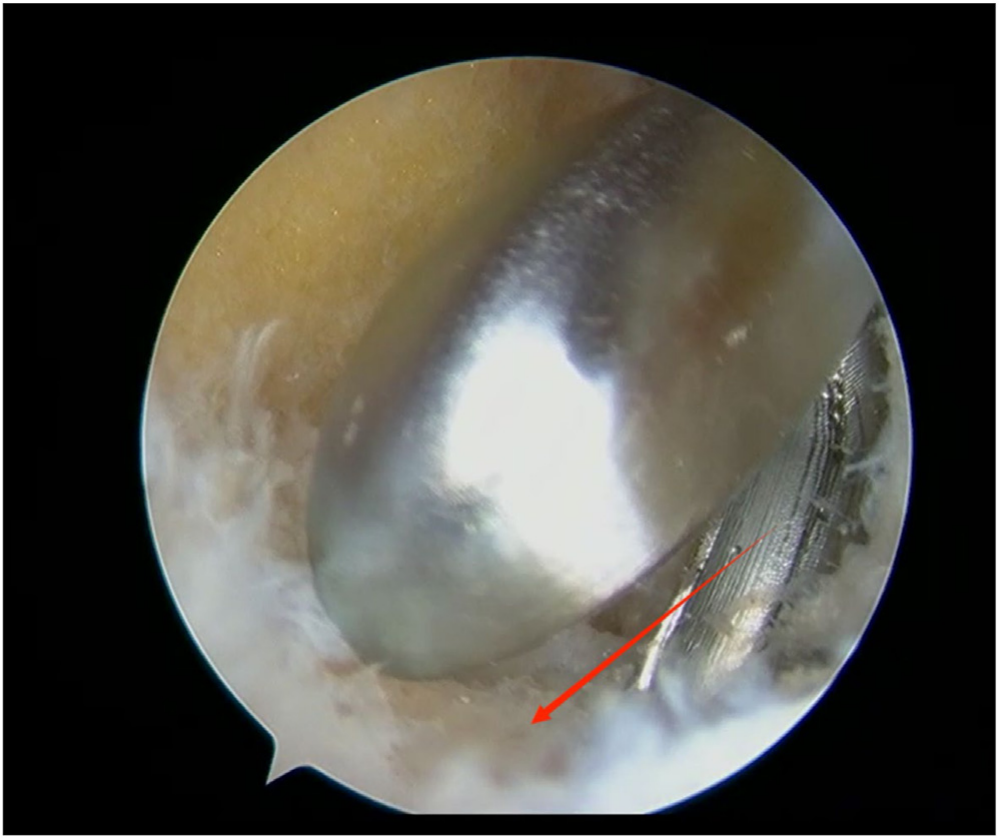
Arthroscopic view of sampling of the diseased patellar tendon and calcifications for pathological examination through the lateral portal to the tibial tubercle. The red arrow points to the diseased patellar tendon and calcifications.

**Fig 10:**
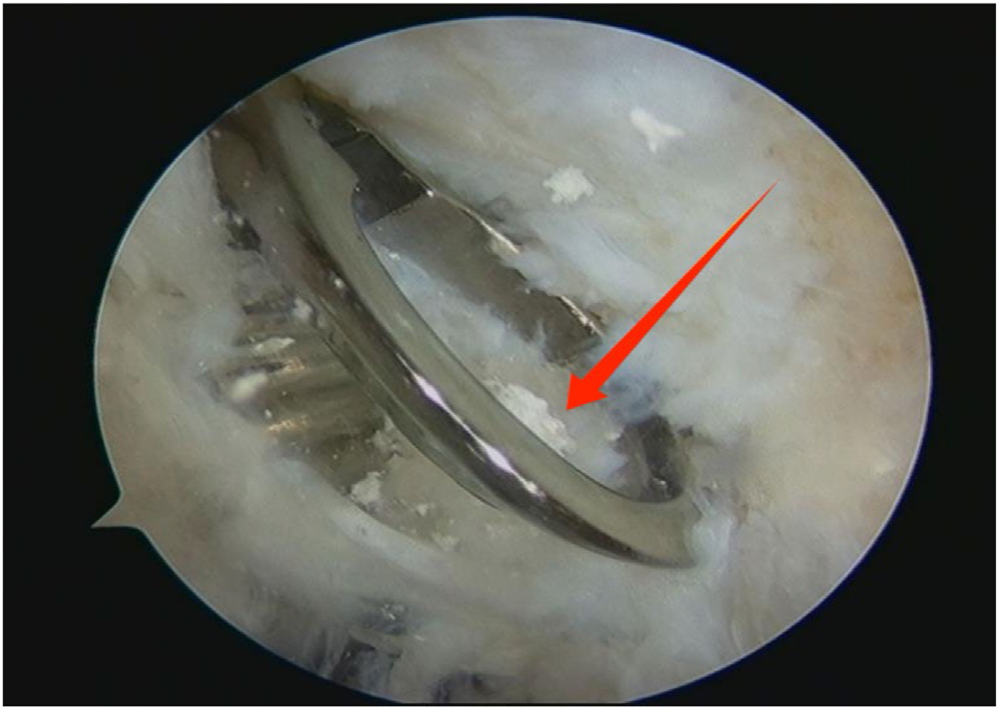
Arthroscopic view of sampling of a small amount of urate crystals for pathological examination through the lateral portal to the tibial tubercle. The red arrow points to the urate crystals.

An arthroscope (4-mm × 30° video arthroscope; Smith & Nephew, Smith & Nephew Surgical Implants) enters through the lateral portal to the tibial tubercle, while the electric shaver (planing blade, teardrop shape, A2-195 × 4.1 × 3.3; Smith & Nephew, Beijing Ruilang Taike Medical Devices Co., Ltd.) enters through the medial portal to the tibial tubercle. Arthroscopic exploration reveals the fat pad above (Fig 5), the tibial tubercle osteophyte, the posterior side of the patellar tendon, and the proximal patellar tendon insertion (Fig 6); this enclosed area is referred to as the “bursa.” The third step is to clean the tibial tubercle osteophyte. Since the tibial tubercle osteophyte is a 3-dimensional structure, the shaver and drill (planing blade, cylindrical, 195 × 5.6 × 4.3 mm; Smith & Nephew, Beijing Ruilang Taike Medical Devices Co., Ltd.) need to clean it from the proximal to the distal end and from the front to the back, using forceps (ZF568Rd20Cr, 180 × 2 × 6 cm; XINGHUA SURGICAL INSTRUMENT) to take a small amount of the tibial tubercle osteophyte as a pathological sample (Fig 7). The cleaning standard is that the protruding part of the tibial tuberosity disappears and a slight depression remains (Fig 8). During this process, care must be taken to ensure that the cleaning reaches the proximal patellar tendon insertion; if it does, further cleaning should be stopped to avoid further damage to the patellar tendon insertion. The fourth step is to clean the calcified lesions on the posterior side of the patellar tendon and at the upper end of the proximal patellar tendon insertion. The shaver is first used to clean the calcification on the posterior side of the patellar tendon. Calcification often appears yellow, while healthy patellar tendons are white. During the debridement of calcification, it is crucial to continuously pay attention not to damage the surrounding white areas. Once the normal patellar tendon tissue deep to the calcification is exposed, further debridement should not be continued. Following this, the calcified lesions on the upper surface of the junction between the patellar tendon and the tibia are cleaned by shaver, and a small amount of diseased patellar tendon and calcified material is taken as a pathological sample (Fig 9). The fifth step is to clean the urate crystals. The shaver is used to clean the deposited urate crystals on the tibial bone bed from proximal to distal, ensuring a slight depression remains at the tibial tuberosity. Next, the urate crystals deposited on the posterior side of the patellar tendon and on the proximal patellar tendon insertion are cleaned, taking care to preserve the patellar tendon tissue and avoid damaging the insertion point. Finally, a sample of the urate crystals is taken for pathological examination (Fig 10). The sixth step involves swapping the positions of the arthroscope and shaver and repeating steps 3 to 5. Last, arthroscopic exploration of the tibial bone bed and the patellar tendon confirms complete osteophyte removal and no tendon injury, followed by irrigation of the surgical area, wound closure, and pressure dressing with sterile gauze and elastic bandage at the wound site. After completion of the surgery, the patient is transferred back to the ward.

### Postoperative Rehabilitation

After the anesthesia wears off, the patient can begin ankle pump exercises and isometric contractions of the quadriceps. Within 24 hours, they can fully weightbear and walk with moderate protection using crutches. Knee flexion training is not restricted; the patient can flex the knee as tolerated within a pain-free range. The patient is expected to regain normal knee flexion angles in about 3 weeks.

Table 1 presents the advantages, disadvantages, and risks of this technique; Table 2 outlines the key points and precautions of this technique; Video 1 demonstrates the entire procedure.

**Table 1:** Advantages, Disadvantages, and Risks

Advantages
This arthroscopic technique typically requires only 2-portal technology.
The approach does not need to enter the joint cavity, allowing for quick, minimally invasive recovery.
It does not damage the infrapatellar fat pad tissue.
Clearing the tibial tubercle osteophyte while simultaneously removing urate crystal deposits may better improve patients’ anterior knee pain symptoms.
Disadvantages
Creating the surgical field requires a high level of expertise; poor cavity creation may affect the surgical field view.
This technique may not be suitable for patients with concomitant intra-articular lesions.
Risks
There may be a risk of patellar tendon insertion injury.
There may be a risk of excessive bone resection of the tibia.

**Table 2:** Key Points and Precautions

Key Points
When clearing calcified areas, care should be taken to protect the patellar tendon insertion site.
Surgeons should aim to position the shaver opening toward the osteophyte during the operation to avoid causing damage to the patellar tendon.
During surgery, switching between the working and viewing ports is necessary to ensure thorough removal of the lesion.
When urate crystal deposits are widely dispersed, efforts should be made to clear them as much as possible to ensure the effectiveness of the surgery.
Try to arthroscopically remove the bone fragments within the patellar tendon as much as possible using the grinding drill and forceps.
Precautions
Accurate preoperative positioning is essential when creating the surgical field to avoid entering the joint cavity and causing damage to intra-articular structures.
When clearing gout crystals, care should be taken to avoid injuring the patellar tendon.

## Discussion

Various surgical methods are available for treating OSD, but arthroscopic tibial tubercle osteophyte debridement is considered the most effective. Compared to open surgery for refractory OSD, it causes less patellar tendon damage, allows for better early postoperative recovery, leaves no incision scars in front of the osteophyte, and provides better cosmetic outcomes.^3^^,^^7^, ^8^, ^9^, ^10^ Our patients were able to bear weight and walk the day after surgery, with no joint adhesions due to our technique not entering the joint space, facilitating easier joint angle recovery postoperatively, and the incisions on the sides of the patellar tendon did not affect knee joint flexion and extension. This demonstrates that arthroscopic osteophyte debridement may offer advantages.

The portal to arthroscopic tibial tubercle osteophyte debridement varies.^3^^,^^7^, ^8^, ^9^, ^10^, ^11^, ^12^ The common portal is the anterior medial and lateral portals, with the advantage of allowing intra-articular exploration and being more readily accepted by sports medicine practitioners as a standard approach. However, this approach’s disadvantages include the potential for damaging intra-articular structures as surgical instruments reach the upper end of the tibia and the risk of excessive removal of the infrapatellar fat pad for better exposure.^7^^,^^8^^,^^10^ We chose the medial and lateral portals within the lesion center without entering the joint space, preserving the infrapatellar fat pad tissue.

We did not use the conventional x-ray–guided needle localization method or the ultrasound-guided technique to ensure complete osteophyte removal.^7^, ^8^, ^9^, ^10^, ^11^ We assessed osteophyte removal arthroscopically, making the surgery faster and reducing radiation exposure for both the surgeon and the patient.

Urate crystal deposition and gout stones are not often detected on MRI, x-ray, or standard CT imaging, so clinicians often discover them through arthroscopic exploration.^13^, ^14^, ^15^ Urate crystals appear chalky white under arthroscopy and are commonly found on tendons, synovium, bones, and cartilage. Identifying urate crystals under arthroscopy may require clinicians to have a certain level of experience, which is crucial. Additionally, whether complete removal of monosodium urate crystals through debridement can be achieved is essential, as it may affect the relief of clinical symptoms in gouty arthritis and improve prognosis. We explored the tibial tubercle and surrounding soft tissues for urate crystal deposits and cleared them under arthroscopy, effectively alleviating anterior knee pain symptoms. Our technique did not enter the joint space but also prevented urate crystal entry into the joint space, preventing intra-articular gout. In conclusion, we believe this technique will offer a new approach for the surgical treatment of OSD complicated with gout in patients with anterior knee pain.

## Disclosures

The authors declare the following financial interests/personal relationships which may be considered as potential competing interests: Z.L. reports financial support was provided by Beijing Municipal Science and Technology Commission. H.H. reports financial support was provided by Beijing Nova Program. Y.Y. reports financial support was provided by National Natural Science Foundation of China. All other authors (L.C., Y.Q., Y.H., Z.H., J.G.) declare that they have no known competing financial interests or personal relationships that could have appeared to influence the work reported in this paper.

## Supplementary Data

Video 1 Preoperative x-rays, computed tomography scans, and magnetic resonance imaging are used to clarify the patient’s diagnosis and assess the location and size of the osteophytes. Considering the patient’s history of gout, urate crystals can be removed arthroscopically. The surgical approach involves a medial and lateral approach to the tibial tuberosity without entering the joint cavity. During the procedure, a shaver and drill are used to remove the tibial tuberosity osteophyte and clear the urate crystals and calcification. It is necessary to alternate the positions of the working and viewing portals during the surgery to ensure a comprehensive exploration and treatment of the affected area. Before concluding the surgery, the proximal patellar tendon insertion should be examined to ensure the integrity of the tendon structure is not compromised.
